# Multidimensional Human Dynamics in Mobile Phone Communications

**DOI:** 10.1371/journal.pone.0103183

**Published:** 2014-07-28

**Authors:** Christian Quadri, Matteo Zignani, Lorenzo Capra, Sabrina Gaito, Gian Paolo Rossi

**Affiliations:** Dept. of Computer Science, University of Milan, Milan, Italy; Umeå University, Sweden

## Abstract

In today's technology-assisted society, social interactions may be expressed through a variety of techno-communication channels, including online social networks, email and mobile phones (calls, text messages). Consequently, a clear grasp of human behavior through the diverse communication media is considered a key factor in understanding the formation of the today's information society. So far, all previous research on user communication behavior has focused on a sole communication activity. In this paper we move forward another step on this research path by performing a multidimensional study of human sociality as an expression of the use of mobile phones. The paper focuses on user temporal communication behavior in the interplay between the two complementary communication media, text messages and phone calls, that represent the bi-dimensional scenario of analysis. Our study provides a theoretical framework for analyzing multidimensional bursts as the most general burst category, that includes one-dimensional bursts as the simplest case, and offers empirical evidence of their nature by following the combined phone call/text message communication patterns of approximately one million people over three-month period. This quantitative approach enables the design of a generative model rooted in the three most significant features of the multidimensional burst - the number of dimensions, prevalence and interleaving degree - able to reproduce the main media usage attitude. The other findings of the paper include a novel multidimensional burst detection algorithm and an insight analysis of the human media selection process.

## Introduction

With the emergence of today's technology-assisted society, human communications and social interactions are often built on top of different techno-communication channels, including online social networks, email and mobile phones (calls, text messages). Human behavior and social interactions through the diverse communication media have become a subject of intensive research because a clear grasp of the same is considered a key factor in understanding the formation of the today's information society. Consequently, a vast and well-established literature regarding the properties of social networks built on each communication media is now available [1]–[5]. More recently, interesting studies about the dynamics of user interactions have also emerged, mostly enabled by the availability of large scale datasets enriched with physical timestamps of events. All these studies [6]–[14] have shown that a pronounced temporal inhomogeneity characterizes this type of communication activity, i.e. users perform sequences of rapidly occurring events, interleaved by long inactive periods. As a consequence, starting from the seminal work of Barabasi [15] who stressed the inappropriateness of the Poisson process in their modeling, human dynamics has become to be considered as bursty.

Previous research on user communication temporal behavior has mainly focused on a sole communication activity. Nonetheless, human sociality is expressed through different communication channels - each channel describes a specific dimension of human sociality as a whole - and therefore the understanding of its dynamics and complexity may be improved by reckoning on all different dimensions together. Thus, the study of multidimensionality has become an inescapable fact when designing both practical and theoretical frameworks that describe human activities. A few seminal works [16]–[19] have adopted a multidimensional approach to study the structural properties of social networks when multiple communication channels are considered, while [20], [21] model a collective bursty temporal process as composed of subprocesses and study spatiotemporal correlations inside utilization patterns of mobile service users.

In this paper we move forward another step on this research path by performing a multidimensional study of human sociality as an expression of the use of mobile phones, where the user has different communication media. While in [22] we explored the structural properties of the mobile multidimensional social network, this work focuses on user temporal communication behavior in the interplay between the two complementary communication media, text messages and phone calls, that represent the bi-dimensional scenario of analysis. Our study provides a theoretical framework for detecting and analyzing multidimensional bursts by introducing a new burst detection algorithm and metrics suitable to describe multidimensionality features. In fact multidimensional bursts exhibit a complex inner structure that accounts for how individuals organize their activities once a burst is initiated. The interplay among the different dimensions can be fully described by defining the triplet of metrics 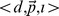 which captures the number of dimensions 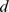, the dimensions prevalence 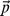 and the tendency to switch among dimensions 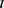. Finally, we propose a multidimensional burst generative model by means of Generalized Stochastic Petri Nets which proves able to reproduce all the above-mentioned features.

The use of this general framework enabled us to offer empirical evidence of multidimensional bursty nature by analyzing the combined phone call/text message communication patterns of approximately one million people over a three-month period. Multidimensional analysis sheds light on human social interactions by phone and give answers to new emerging research questions: How do individuals schedule different phone activities? Is the mental selection process driven by technology or by social relationships? We find that the structure of the phone activity sequences is mostly influenced by the communication media. This behavior has to do with the uncomfortable and time consuming task of media switching which is able to condition the mental scheduling process.

## Materials and Methods

### Dataset

We use a large anonymized dataset of mobile-phone Call Data Records (CDR) - containing call and text message activities of mobile subscribers - gathered in a large European metropolitan area from March 26 to May 31, 2012, for a total of 67 days. The dataset contains in all more than 69 million phone call records and 20 million text message ones. Each record contains the following information: date, hour, source user-ID, destination user-ID and, re phone conversations, duration of the call in seconds.

The dataset was preprocessed to obtain significant time series of outgoing phone activities to highlight the active role in the initialization of the communication. The preprocessing phase focused on the dynamics of per-day activities of users. In fact, in line with the arguments proposed in [23], we are less interested in observing the role of human circadian and weekly activity patterns which act on both weekly and 24-hour time scales. In practice, we put more emphasis on human behavior in performing phone activities during a day than in natural life cycles.

The following analysis considers the outgoing phone activities of individuals as a sequence of discrete temporal events. For each pair (user, day), we build the time series of the outgoing phone activities performed by that particular user on that particular day. Finally, we obtain the set of time series representing our sample. Thus, an event time series 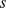 of a given pair (user, day) is defined as: 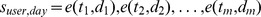, where 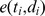 is the 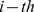 event, an outgoing call or text message activity performed by 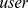 at 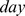, 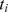 represents the event's starting time and 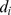 is its duration, which we assume to be 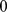 in case of text message. From now on, we will use the terms time series and sequences as synonymous with 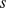, disregarding indexes.

For this paper's purposes, we only considered the time series having a relevant number of both texts and calls, i.e. lying above a given threshold. This led us to obtain two sets of time series, namely *D 1* and *D 2*, by selecting two values of the threshold for outgoing calls and texts: for D 1 the threshold is 25, while for D 2 the threshold is 10. Moreover, in order to exclude anomalous users like robot-based event generators, telecom frauds, telephone sales, and such, we required that the daily activities should not exceed the threshold of 100 calls and 200 texts. After this preprocessing, we found D 1 to contain 5,716 time series and D 2 to contain 134,736. This way D 1 accounts for time series expressing a very intense activity, while D 2 weakens the activity level allowing us to check and generalize the behaviors observed in D 1 [24].

### Burstiness

It is commonly accepted that inhomogeneous time-dependencies within a sequence of discrete events show a heavy-tailed inter-event time distribution, where the inter-event time is the elapsed time between two consecutive events in a time series 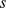. More precisely, in our case inter-event times are defined as 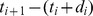 - while for text messages it is simply 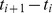.

To confirm the bursty nature of phone activities, when considering both texts and calls, we apply to D 1 and D 2 the method proposed in Vasquez *et. al*. [25] and extended by McGlohon et. al. [26]. We fit the inter-event time data by using MLE (Maximum Likelihood Estimator) for exponential and Zeta distribution, and select as best model the one that minimizes the AIC (Akaike Information Criterion).

### Burst detection algorithm

Given a time series it is important to correctly identify which events are performed in a burst train and which are not. To this end different algorithms are developed to automatically find out bursts and they differ in the features they consider to identify the bursts: variation of event arrival rate [27], number of events occurred in a specific time window [28], [29] and inter-event time threshold [23], [30], [31]. In mobile phone dataset analysis the most used approach is the inter-event time threshold. This approach defines a burst as a group of consecutive events having inter-event time below a certain threshold. Nevertheless all these approaches are limited to one-dimensional case and do not consider temporal overlapping.

If we move from one dimension to a multidimensional burst time series the burst detection algorithm based on inter-event time of consecutive events is unable to correctly identify bursts, as they might be affected by a temporal overlapping between different dimensions. In fact, circumstances may exist where people communicate and socialize by different media simultaneously, as typing text messages while talking with friends nearby. Albeit rare, we observe this behavior in a few sequences of our datasets, where texts were interspersed with calls. Here we propose a different approach that is viable for any multidimensional case.

Let us start by providing a formal definition of burst. In a burst each event, except for the first and last ones, has at least 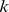 neighbor events within 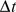. Let us consider the event time series 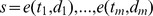 and the function 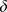 representing the time elapsed between two events 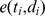 and 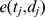 defined as follows:
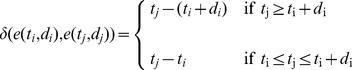
(1)


For each event 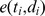 of the sequence, we consider the set 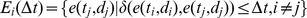, which represents the set of all events 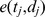 having a time gap from 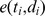 below or equals to 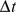.

We define a *burst* as a sequence 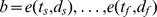 such that each set 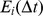, with 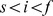, has cardinality greater or equals to 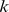. According to this definition the burst length 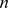, defined as 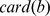, is 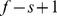.

**Figure 1:**
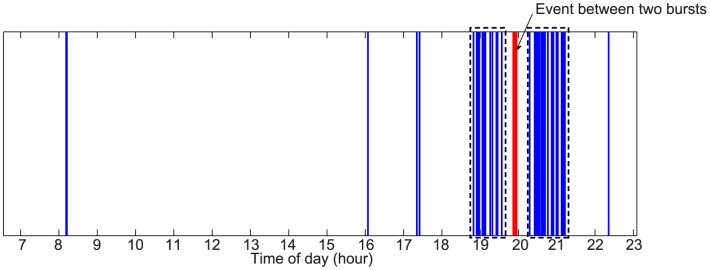
Example of event time series. This example explains how the burst detection algorithm works. The algorithm correctly identifies the densest regions (black dashed boxes) avoiding to include the event in between (red solid line).

From an algorithmic point of view, the given definition, and in general the idea of burst, can be reported in a way that fits with the model adopted by the density-based clustering algorithm framework [32]. A burst can be seen as a time period where the probability density function 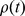 exceeds a prefixed threshold. Density-based clustering is exactly a non parametric framework whose aim is the extraction of high density regions of 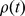. Therefore we assume that the points we group correspond to the time of the event 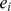 which comes from an unknown probability density distribution 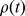, while bursts are the clusters. Among the various density-based methods proposed in the literature, we select DBSCAN [33] because it scales to a large dataset and is robust against noise. Given a distance threshold 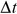, and a threshold 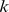 on the number of the events within the interval 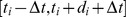 (the choice of the interval 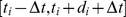 in the construction of the neighborhood set accounts for the asymmetry of the function 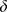), the algorithm finds the maximally connected component (in terms of density reachability) of events at a distance smaller than 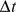 from some core points. By the term core point, we mean a point 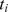 such that the number of events in 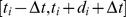 is greater than 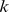. In Figure 1 we illustrate how the algorithm operates on a toy sequence. In the figure we also observe an advantage that the density-based approach offers w.r.t. simple aggregation on the inter-event time [23]. Density-based methods overcome the so-called ‘chaining-effect’ which affects the single linkage methods as the inter-event aggregation. In fact the sole aggregation can result in different clusters merged by a ‘chain’ of single points between the clusters. In the figure the chaining-effect induces the two detected bursts to merge into one due to the single event in between.

### Multidimensionality features

In this section we focus on the characterization of multidimensional bursts as the very general burst category that includes one-dimensional bursts as the simplest case. In the following we omit the features that have been already defined in one-dimensional burst analysis and that are still valid in the general case (such as inter-times or burst length). Therefore we focus on a set of features, and corresponding quantifying metrics, able to fully characterize multidimensionality. Indeed, multidimensionality features each single burst, not the overall time series, and so models and metrics relative to burst sequence still hold. Here we zoom in the single burst structure to describe its inner multidimensionality.

To this end, first we introduce the variable 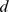 that indicates how many different types of activity exist within a burst, *i.e*. the number of its dimensions, and denote as 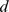-burst a 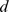-dimensional burst. We can now represent a 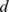-burst as a sequence of symbols belonging to the alphabet 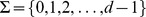, where 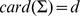. In our bi-dimensional datasets, we code *text message* = 1 and 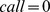 and represent bursts as binary sequences.

Secondly, we characterize the burst multidimensionality by considering the relative importance of the activities inside the burst. Given a 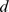-burst, we define as *prevalence* the vector 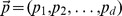 where 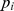 is the probability to draw the 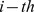 dimension symbol when considering a multinomial process on the burst: 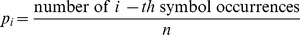, where 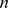 is the burst length.

In our case, 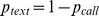 holds and measures the *prevalence* of one of the two activities w.r.t. the other, accounting for the media selection preferences performed by a given user in a given burst of phone activity.

Nevertheless, these two features fail to describe how often a user switches from one medium to the other. Let us consider, for example text/call bursts. In addition to the one-dimensional burst, where the user decides to perform all activities on a single medium, multidimensional burst is a multiple symbol sequence. Symbols can be more or less interleaved inside a sequence, accounting for how often the user switches between media inside a burst. This way switches divide a burst into sub-sequences, each being a sequence of a sole symbol. An extreme case, very similar to the one-dimensional one, occurs when the burst can be divided in exactly two sub-sequences, one containing text messaging, the other call only. We name this burst a *disjoint* burst, as the user is definitely separating the two media. Single and disjoint bursts clearly account for a monotone behavior w.r.t selecting a particular type of activity; for example, a user may decide to use only one communication medium or to send all texts prior to performing other activities.

By contrast, bursts where symbols of different media are interleaved with one another are clearly an observable effect of the multidimensionality and can provide valuable insight into the selection process underlying the user's activities. For purposes of clarity, we use the term *interleaved* to identify bursts that are neither disjoint nor one-dimensional. Of course, *interleaved* bursts exhibit different degrees of interleaving, which account for how often the user changes media or, equivalently, how many sub-sequences exist in the symbol sequence.

Intuitively, the higher the degree of interleaving, the farther the sequence moves away from the binary sequence representing the one-dimensional or disjoint burst. In the general case of a 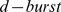 the interleaving degree of a burst is formally expressed as follows:
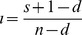
where 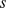 is the number of switches from one symbol to another inside the sequence. The numerator represents the difference between the number of sub-sequences inside the current burst, 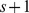, and inside a generic disjoint type burst, 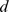. The denominator is a normalization factor accounting for the difference between the number of events in the burst, 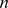, and the minimum number of events of a 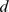-dimensional burst, 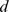. This coefficient assumes values in the interval 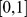: 0 means that the burst is a disjoint or one-dimensional burst, while 1 means that the type of activity changes from event to event. For example, let us consider the bursts having the following events sequence:

**Table:** 

Burst	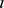
0000011111	0
0011110001	0.25
0011001100	0.375
0101010000	0.625
0101010101	1

Finally, the triplet 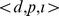 fully describes the multidimensional nature of a burst.

As a final step of the multidimensional analysis, the comparison between the dataset's time series and a randomized dataset (null model) is the mechanism we adopt to see if the footprint of human behavior can be recognized in a user's sequence of events or if it is simply the consequence of a random selection of communication media. To produce the randomized dataset, we shuffle the user activities of the original dataset as follows: we start with the time series of activities and randomly permute the order of text messages and phone calls a user executes. This shuffling method allows us to leave untouched the inter-event times, ergo the detected bursts as well, while removing the activity type selection process, which in our case corresponds to the selection of communication media.

## Results and Discussion

### Multidimensional burst features

The empirical analysis of the mobile phone activities of the described datasets shows a clear power-law behavior of inter-times distribution as confirmed by Table 1 where around 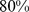 of time series in both datasets are better modeled by a heavy-tail distribution.

**Table 1:** Fitting results.

D 1		D 2	
Power law	Exp.	Power law	Exp.
87.16%	12.84%	76.67%	23.33%

Percentage of time series, in D 1 and D 2, which are best fitted by the Zeta (power-law) and exponential distributions. The best model selection has been performed according to Akaike Information Criterion.

### Bursts

**Figure 2:**
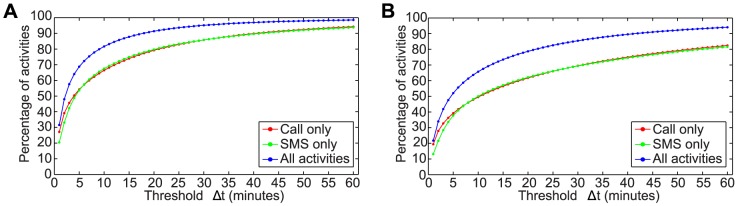
Burst detection results. Mean of the percentage of events inside the bursts by varying the threshold 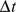 from 1 to 60 seconds: (a) D 1, (b) D 2.

In Figure 2 we show the results of the burst detection algorithm applied by varying the threshold 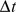 from 1 to 60 minutes and 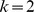. The latter value enables the comparison of our results with those obtained by implementing the single linkage methods. In both datasets we found that phone activities are very bursty and, even when we execute the detection algorithm with the tight threshold value of 10 minutes, more than 80% of activities occur inside burst. In the same figure we also report the results we obtained by considering texts and calls separately. In both one-dimensional cases these percentages are much lower due to the fragmentation of bursts when the two media are alternated. For the same reason, as shown in Table 2, the burst length too definitively increases when the scenario is enriched by more dimensions.

**Table 2:** Burst length statistics.

	D 1			D 2		
	Mean	Med.	Std.	Mean	Med.	Std.
**Call**	8.2	6.0	7.4	6.2	5.0	5.1
**Text**	8.9	6.0	9.3	6.2	4.0	6.1
**Phone**	13.8	9.0	14.7	8.2	6.0	7.9

Comparison of burst length (mean, median, and standard deviation) among the one-dimensional cases and the multidimensional one.

**Figure 3:**
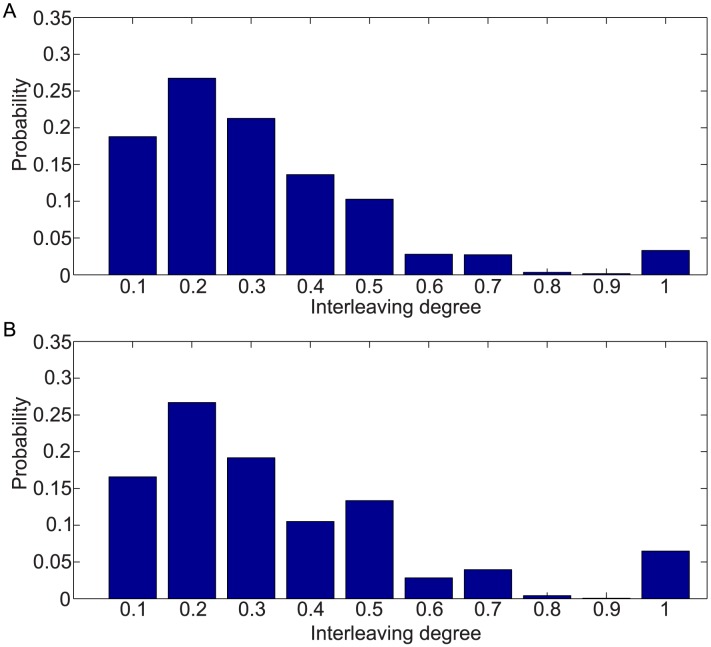
Histogram of interleaved degree. Interleaving degree computed on interleaved burst. (a) D1: mean 0.32, median 0.29, and standard deviation 0.20. (b) D2: mean 0.36, median 0.31, and standard deviation 0.23.

In Table 3 we report the comparative analysis between original and shuffled time series, along with the percentage of variation rate (defined as the difference between the shuffled value and original value divided by the original value). We can observe that the shuffled process causes a reduction of one-dimensional and disjoint burst types; in particular, we see a significant decrease in one-dimensional bursts. In fact, about 50% of bursts are one-dimensional or disjoint, unlike what we would observe if the choice were obtained randomly. Moreover, the overall degree of interleaving is very low. In Figure 3 we report the histogram of the values of the interleaving degree computed on the interleaved burst type only. The results show a low coefficient value, more than 80% are below or equal to 0.5, accounting for a very low level of interleaving attitude.

**Table 3:** Comparative analysis between original and shuffled time series.

	D1			D2		
Burst type	Original	Shuffled	Variation rate	Original	Shuffled	Variation rate
One-dimensional	24.57%	6.42%	−73.86%	32.79%	10.72%	−67.29%
Disjoint	18.37%	14.49%	−21.12%	25.11%	21.51%	−14.33%
Interleaved	57.06%	79.08%	+38.60%	42.10%	67.77%	+60.95%

Comparative analysis between original and shuffled time series for each dataset. The shuffled time series was obtained from the original ones by performing a random permutation of the order of text messages and phone calls.

The aforementioned arguments enforce the hypothesis that the execution order of phone activities is mainly affected by the need to minimize the switching overhead between different communication media (and the relevant apps). People experience a certain inertia which makes them to lean in single dimension and to persist in there up to completion of the planned burst activities.

These results magnify the burst nature of human communication and highlight the extent to which the multidimensional approach enriches the big picture of mobile phone communication.

### Social or media driven?

In the previous section we have shown that the processing sequences of human mobile phone activities are likely to be heavily influenced by media selection. This argument is slightly counterintuitive - we would expect human interactions to reflect the personal value (ranking) that individuals ascribe to their social relationships - and thus deserves further analysis. Provided that individuals have the attitude to organize their sequences of phone activities in multidimensional bursts with a very low interleaving degree, the question to answer is: does the order in which activities inside a multidimensional burst reflect the ranking that the user ascribes to his/her social relationships?

To provide a quantitative answer to this question, we perform a correlation analysis between burst activities and sociality rankings.

The burst activities ranking has been performed as follows. We consider the sequence 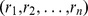 where 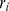 represents the receiver of the 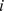-th phone activity performed by user in a burst and we consider the vector given by the first occurrence of all the receivers. The burst rank of each receiver 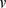 is defined as the index of 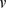 in the vector of the first occurrences. For example consider the following sequence (b, a, a, b, a, c, c, b). The vector of the first occurrences is 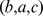 which gives the following ranking 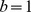, 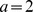 and 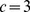. We can stretch the scope of this ranking notion from a single burst to a full day by joining all the sequences corresponding to the bursts happened on a specific day.

We can describe the sociality ranking by organizing, in decreasing order, the neighbors of each user on the basis of the number of interactions the user has performed with his/her neighbor. Here we adopt the term ‘interaction’ to indicate a voice call or text message issued by a user. The sociality ranking has been shortened by removing neighbors not included in the burst/day ranking. This way the two rankings have the same cardinality. The evaluation of the Spearman correlation coefficient enables the comparative analysis of the two rankings.

**Figure 4:**
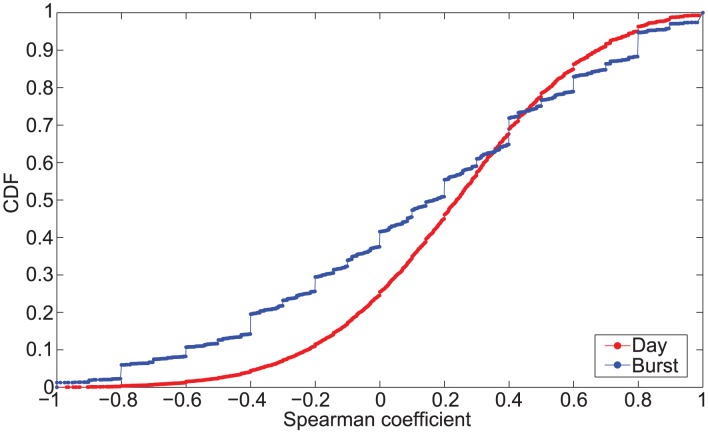
Distribution of Spearman correlation coefficient. CDF of the Spearman correlation coefficient computed on D 2 considering sequences with more than three distinct receivers. We report the distribution for both day (mean 0.21, median 0.24 and std 0.36) and burst (mean 0.12, median 0.17 and std 0.50) level.

**Figure 5:**
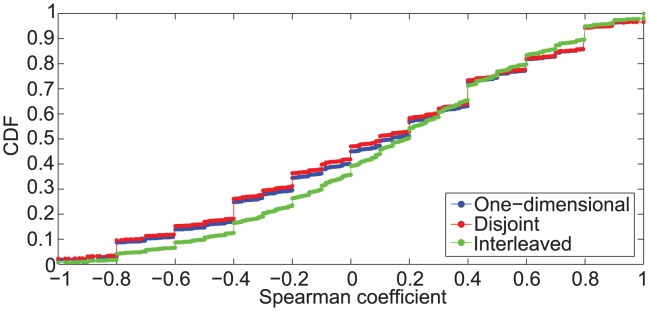
Distribution of Spearman correlation coefficient per burst type. CDF of the Spearman correlation coefficient computed on D 2 by grouping burst by type and considering sequences with more than three distinct receivers.

We analyze the distribution of the Spearman coefficient 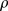 at both day and burst level and we report the relevant distributions in Figure 4. To avoid artifacts induced by small number of points, we consider sequences with more than three different receivers. At a burst level we have nearly 28% of bursts showing high degree of correlation or anti-correlation (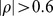), while at day level we observe this percentage decreasing to 15%. These results highlight that the choice of the next activity is not driven by social importance. This still holds even in case of more regular behavior in terms of media selection, as it happens in one-dimentional or disjoint bursts. As we can see in Figure 5, where the CDF of the Spearman coefficient grouped by burst type is reported, there is no significant difference between burst types. In fact, we observe that around 30% of one-dimensional and disjoint bursts show high degree of correlation or anti-correlation w.r.t. 25% of interleaved bursts.

We can conclude that the communication medium is the main ingredient to determine the organization of our sequence of activities inside a burst.

### A generative model of two-dimension bursts

Next we present a generative model for two-dimensional bursts, along with results from a comparison between selected indexes and statistics regarding an excerpt of sample data and a combinatorial analysis of binary sequences.

The model aims at representing single bursts only, yet it is parametric in burst length (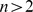), thus it might be easily interfaced with upper layers which provide values for 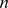 (e.g., by drawing from a given distribution) and model inter-burst times.

**Figure 6:**
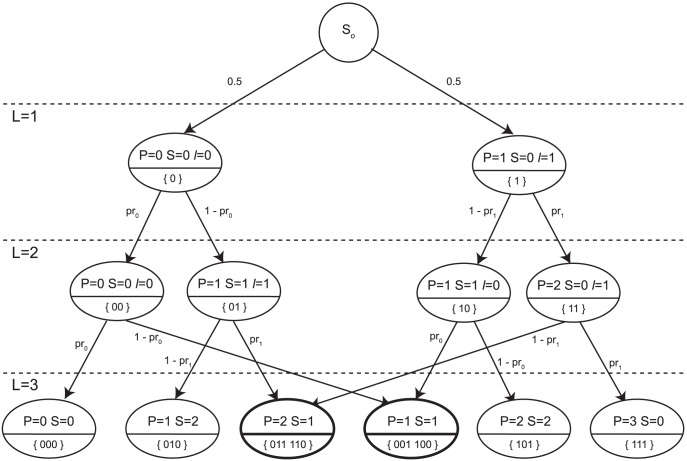
Discrete time Markov chain. Graphical representation of the discrete time Markov Chain (MC) in case of burst length 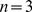. MC states are described by four integer variables, 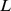 (length), 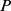 (prevalence), 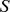 (switches) and 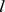 (last). 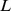 denotes the length of (sub)sequences represented by a state (in the picture values of 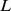 correspond to the depth level in the DAG – Direct Acyclic Graph). 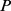 and 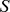 indicate the number of ‘1’s and the number of switches, respectively. 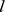 denotes the last occurred event and allows tracking switches. Each state, but 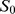, is annotated with some variables' bindings (top) and the (sub)sequences it represents (bottom). States drawn in bold correspond to aggregates of sequences. The parameters 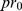 and 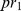 represent the probabilities that the next event coincides with the last one. In order to make the MC irreducible, we assume that each final state (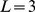) brings the system back to state 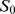 with probability 1 (these connections are omitted in the picture).

The generative model of bursts of given length builds on a discrete time Markov Chain (MC), which is instantiated in Figure 6 for the simplest case, 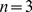. Its regular structure makes it possible to infer the MC appearance for any 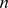. Each MC state represents a (set of) (sub)sequence(s), and may be described by bindings of some integer variables, 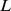 (length), 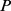 (prevalence), 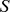 (switches) and 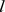 (last), that take values in 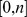, 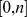, 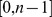 and 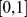, respectively. In Figure 6 states are annotated with characterizing bindings. The value of 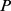 indicates the number of ‘1’ in (sub)sequences. As for 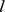, it denotes the last event that occurred and allows one to track switches. The one step transition probability matrix of the MC builds on two parameters, 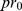, 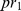, which define the probabilities that the next event in a (sub)sequence coincides with the last one. The values 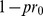, 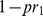, thus stand for switch probabilities. We assume that at the beginning of a burst the two possible events occur with same probability.

Some MC states, drawn in bold, correspond to aggregates of (sub)sequences: the higher 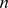, the bigger the impact of aggregation. This way the complexity of the generative model's solution, in terms of number of states, drops from exponential (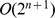) to polynomial (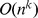, 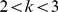), so enabling the analysis of bursts of realistic size.

However, a direct use of the MC is unfeasible because its size become relevant even for small values of 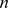. The need of identifying a more expressive formalization of the generative model of bi-dimensional bursts as binary sequences led us to consider a timed extension of Petri Nets (PN), known as Generalized Stochastic Petri nets (GSPN) [34], [35]. The GSPN formalism, briefly introduced in the Appendix, eases the task of modelers by providing compact, parametric and stochastically-reconfigurable representations of even huge Markov processes.

The fact that a GSPN maps to a Markov process, i.e., a Continuous Time MC (CTMC), does not represent a problem. If we consider only the instants at which the state of the system changes, and we number these instances 0, 1, 2,.., then we get a discrete time Markov chain that is called Embedded Markov Chain (EMC), or “jump process”, of the CTMC. Letting 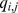 be the exponential transition rate from state 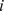 to state 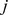 of a CTMC, the one-step transition probability matrix of the EMC is defined by setting 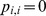, 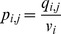 if 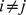, where 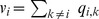. The stationary probability distribution 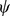 of the EMC (which exists and is unique if the EMC is irreducible/positive recurrent) can be computed from that of the CTMC (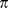), and vice-versa: 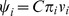, where 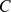 is a normalizing constant.

A GSPN generative model parametric in bursts' length was built ensuring that its embedded MC exactly matches the blueprint instantiated in Figure 6 for the case 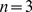.

### GSPN model

**Figure 7:**
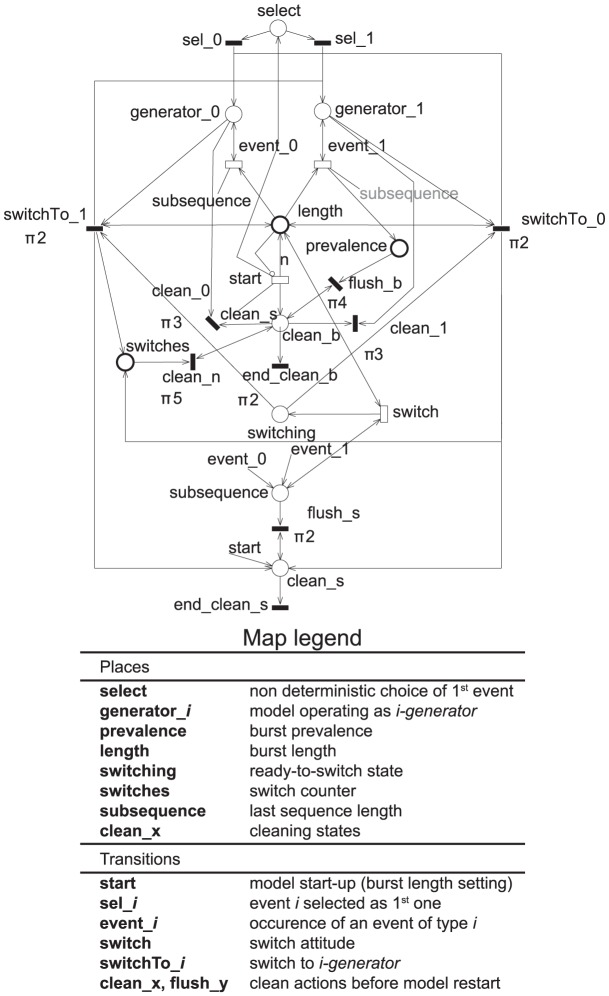
Bi-dimensional burst generative GSPN-model.

The bi-dimensional bursts' generator is depicted in Figure 7, together with an accompanying map legend. The model was edited and analysed using the the GreatSPN package [36], which natively supports GSPN. The model is available at [24] in GreatSPN legacy format. Let us just overview its blueprint.

The model implements two event generators that operate in mutual exclusion. Passing from one generator to the other is triggered by a (symmetric) transition which emulates in some sense the switch attitude of humans.

Transition start starts the model up by putting 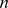 tokens in place length, then (once all of them have been consumed, i.e., 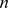 events have occurred) brings the model back to its initial state by triggering a sequence of immediate transitions (recognizable from prefixes clean, flush) clearing the contents of prevalence and other significant places. This way the underlying CTMC is ergodic.

Bursts are randomly built by a pair of mutually exclusive timed transitions (event_0, event_1), one for either type of event (sms/call). The rates of the associated exponential distributions, 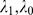, may be interpreted as mean frequencies of events of a given type. The ratio 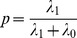 expresses the “average prevalence”, and is one of the model's two stochastic parameters. The switching process is explicitly represented, so as to closely adhere to human dynamics: timed transition switch indirectly conflicts with both transitions event 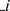, its firing enables one of two (conflicting) immediate transitions (switchTo 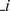) that concretely cause the alternation of the 0 and 1 generators. Places switches and subsequence hold the number of switches occurred since the beginning of the burst, and the length of the last subsequence, respectively. The contents of place subsequence are cleared just after a switch occurrence. Switching is driven by switch's rate, 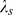, the ratio 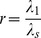 represents the other stochastic parameter of the model.

The parameters 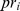, 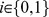, of the EMC associated with the GSPN model are 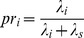. The metrics of interest are derived from the probability distribution on the subset of states 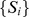 in which 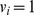 (corresponding to GSPN markings enabling uniquely transition restart, that map to the EMC states in which 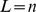). Hence, they can be directly obtained from the GSPN stationary distribution. Referring to the Figure 6, these probabilities may be seen as the probabilities of reaching the final states starting from 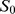.

### Results

In order to validate the model we carried steady state analysis out for a number of configurations. The metrics of interest are the distributions of prevalence and switches (expressed as interleaving degree), which correspond to the distribution of tokens in the homonym places of the GSPN model. Steady-state analysis outcomes are then faced to real data. Besides, we deepen how the model performs against a fully random approach relying on sequence combinatorial analysis.

Note that in case of balanced sequences of length 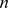, the formulas for the distribution of prevalence (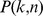, i.e., the probability to draw 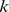 symbols ‘1’) and switches (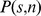, i.e., the probability to draw 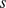 switches) are:
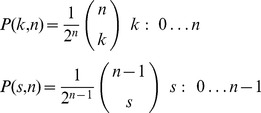



The formula for switches is derived from the following consideration. For a sequence of length 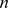, there are at most 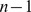 switches. Then the binomial 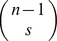 is the number of times in which it is possible to put 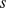 switching points among the 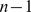 places.

The search parameter space is given by 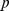 ranging over 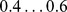, 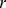 ranging over 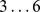, which are symmetric intervals around the mean value observed in dataset, and 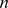 ranging over 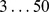. In order to reproduce real prevalence and the interleaving degree distributions, first we selected the best parameters' model using the Kolmogorov distance; then we weighted the obtained per length distributions according to the burst length distribution observed in the datasets.

**Figure 8:**
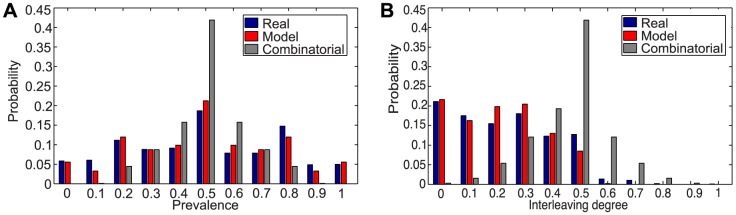
Comparative analysis in case of burst length 13. (a) Prevalence distributions. (b) Interleaving degree distribution. We use the following parameter values: 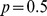 and 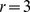.

**Figure 9:**
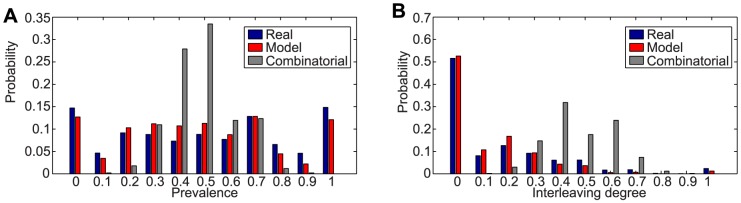
Comparative analysis on dataset D 1. (a) Prevalence distribution. (b) Interleaving degree distributions. We use the following parameters values: 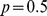 and 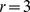.

In Figure 8 and 9 we report the prevalence and interleaving degree distributions of the best fitting model versus the observed and combinatorial ones, both on specific size sequences and aggregated case. There is some evidence that the model fits in sample data much better than combinatorial analysis, which exhibits an unrealistic symmetrical trend with center (approximately) 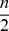. Note that the model is also able to reproduce the peaks due to the one-dimensional and disjoint bursts, which represent the behavior farther from randomness.

The key factor which makes the model closer to the real process than the combinatorial approach is its being driven by an explicit switching process able to reproduce the real behavior. Conversely, the random approach has the prevalence as unique parameter driving the process, switching being just an indirect, not tunable, consequence.

## Conclusions

This paper takes a a step forward in the study of multidimensional human dynamics on networks. Although until now several one-dimensional studies have shed light on important aspects of human dynamics, their extension to a multidimensional approach brings to light key issues that have remained hidden for a long time. This work describes a theoretical framework to address multidimensional human dynamics by providing a multidimensional burst detection algorithm, a set of metrics to characterize multidimensional behaviors and a multidimensional generative model of events. The empirical evidence and validation of the effectiveness of this framework is obtained by following the combined phone call/text message communication patterns of approximately one million people over three months period. While confirming and magnifying the bursty nature of human dynamics, multidimensionality enables to understand the mechanisms underlying the human mental scheduling process whenever people have many different channels available for communication. The findings of this research offer a first contribution to address multidimensional dynamics and open the way to further extensive research. Indeed, the theoretical framework should be embedded in timing burst generative model and extended to networks with more than two dimensions. The multidimensional approach is finally expected to bring about many implications from an application viewpoint. In fact, it may lead to a better understanding of the overall human social dynamics in online social networks and enable the design of novel information spreading algorithms and of autonomic hubs of service on mobile devices.

### GSPN formalism overview

Introduced in the sixties, PN are particular directed bipartite graphs suitable for specifying distributed discrete-event systems. A GSPN is formally a 7-tuple 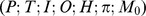, where 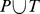 are the nodes of the graph. The elements of 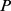, called places and drawn as circles, represent the system's state in a modular fashion. 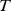 is the set of transitions (drawn as bars), representing actions, events, operations. Connections between places and transitions are defined in matrix form by input, output and inhibitor arc-functions 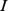, 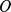, 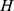: 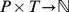, drawn as arrows and rounded head edges, respectively. PN incorporate a distributed state notion, called marking, a map 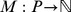. A marking is represented by inscribing place 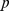 with 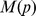 black tokens. 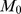 denotes the initial marking of the net. Function 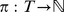 assigns priorities to transitions. Let 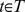: 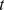 is said to be enabled in 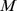 if 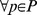 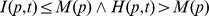, and there are no higher priority transitions meeting this condition. If 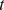 is enabled in 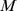 it may fire leading to 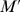, 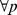, 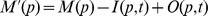. The notation 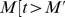 is used. The set of transitions enabled in 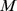 is denoted with 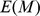. Two enabled transitions are said to be in conflict in 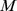 if the firing of one disables the other. Starting from 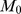 it is possible to build the Reachability Graph (RG) of a GSPN model, whose nodes 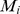 are the markings which are reachable through any firing sequence 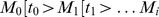, and whose arcs are labelled with the transitions that lead from one marking to another. GSPN include time specifications, so that performance analysis is possible besides classical PN analysis, like structural techniques or state-space exploration. This extension amounts to adding a function 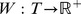 to GSPN's definition. Transitions 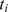 such that 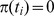 are associated with a random firing delay whose density function is a negative exponential with rate 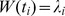. These transitions are called *timed*, their semantics is described by a race model: when a marking enables several (conflicting and/or concurrent) timed transitions, the activities they represent are assumed to run in parallel, so that the state change is due to the transition whose sampled firing delay is minimum. The probability that 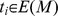 fires is 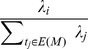.

Transitions 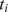 such that 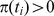 instead represent logical or time negligible actions. They are called *immediate*, because fire in zero time and with priority over timed ones. Graphically they are represented as black tiny bars to be distinguished from timed ones. The value 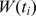 for an immediate transition specifies a weight: if there are several conflicting immediate transitions in 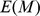 the selection of the one that fires is done using weights 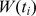, normalized in such a way as to obtain a discrete distribution function. Marking-dependent transition rates/weights may be set, in that case the notation 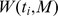 will be used. The RG of a GSPN model contains two different types of nodes: *tangible* markings, in which no immediate transitions are enabled (and therefore the system spends time), and *vanishing* markings, in which at least one immediate transition is enabled (and therefore the time spent by the system is equal to zero). Due to the memoryless property of the exponential distribution, a GSPN whose RG doesn't contain any vanishing loops is isomorphic to a continuous-time Markov chain (CTMC) whose states correspond to the tangible markings of the RG [35]. The component 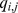 of the infinitesimal generator is the sum of rates of transitions that lead from marking 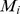 to 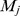 either directly, or via some (suitably weighted) vanishing paths.

Performance indices such as, for example, transition throughputs and the distribution of tokens in a place can be computed on a GSPN model. They are derived from either the transient or the steady state probability vector of the associated CTMC.
